# Colorimetric Sensing of Gram-Negative and Gram-Positive Bacteria Using 4-Mercaptophenylboronic Acid-Functionalized Gold Nanoparticles in the Presence of Polyethylene Glycol

**DOI:** 10.1021/acsomega.3c01205

**Published:** 2023-03-30

**Authors:** Pinyapat Amornwairat, Dakrong Pissuwan

**Affiliations:** † Materials and Engineering Graduate Program, Faculty of Science, Mahidol University, Rama VI Road, Ratchathewi, Payathai, Bangkok 10400, Thailand; ‡ Nanobiotechnology and Nanobiomaterials Research Laboratory, School of Materials Science and Innovation, Faculty of Science, Mahidol University, Rama VI Road, Ratchathewi, Payathai, Bangkok 10400, Thailand

## Abstract

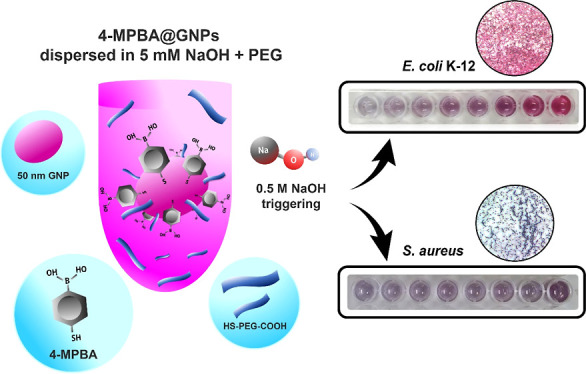

Gold nanoparticles (GNPs) have been used as detection probes for rapid and sensitive detection of various analytes, including bacteria. Here, we demonstrate a simple strategy for bacterial detection using GNPs functionalized with 4-mercaptophenylboronic acid (4-MPBA). 4-MPBA can interact with peptidoglycan or lipopolysaccharides present in bacterial organelles. After the addition of a high concentration of sodium hydroxide (NaOH), the functionalization of the surface of 50 nm GNPs with 4-MPBA (4-MPBA@GNPs) in the presence of polyethylene glycol results in a color change because of the aggregation of 4-MPBA@GNPs. This color change is dependent on the amount of bacteria present in the tested samples. *Escherichia coli* (*E. coli*) K-12 and *Staphylococcus aureus* (*S. aureus*) are used as Gram-negative and Gram-positive bacterial models, respectively. The color change can be detected within an hour by the naked eye. A linear relationship is observed between bacterial concentrations and the absorbance intensity at 533 nm; *R*^2^ values of 0.9152 and 0.8185 are obtained for *E. coli* K-12 and *S. aureus*, respectively. The limit of detection of *E. coli* K-12 is ∼2.38 × 10^2^ CFU mL^–1^ and that of *S. aureus* is ∼4.77 × 10^3^ CFU mL^–1^. This study provides a promising approach for the rapid detection of target Gram-negative and Gram-positive bacteria.

## Introduction

Bacterial contamination has become a major threat to human health. The consumption of contaminated food or water can lead to foodborne and waterborne diseases. The distribution of pathogenic bacteria in the environment, particularly in areas with poor sanitation, is another risk to human health. Disease-causing bacteria can be classified as Gram-negative or Gram-positive bacteria. Bacterial infection is typically characterized by symptoms such as nausea, vomiting, abdominal cramping, and diarrhea, which are caused by enterotoxins, cytotoxins, or plasmid-mediated virulence factors of bacteria.^1−3^ Outbreaks of bacterial infection can lead to severe health complications or fatalities.

Bacterial culture techniques have been commonly used to detect bacteria. However, these techniques are time-consuming, requiring a few days or even a week to provide results. This leads to a delay in bacterial diagnosis, which in turn impacts bacterial distribution control. The rapid spread of pathogenic bacteria should be controlled to protect humans from infection. Therefore, a rapid and affordable bacterial detection approach needs to be developed. The colorimetric technique is an easy visual detection method that does not require the use of expensive instrumentation for detecting bacteria. This technique has become an active approach for bacterial detection.

With advancements in nanotechnology, metal nanomaterials have attracted increasing attention for use in colorimetric techniques.^3^ Gold nanoparticles (GNPs) are considered to be promising materials for colorimetric detection owing to their unique optical properties that provide a broad color range from red to blue. The color of GNPs in a solution mainly depends on their size and shape. These parameters enable GNPs to have their own specific plasmon absorption.^4−7^ The distance between particles also affects the color of GNPs dispersed in a solution. For example, if the distance between particles is less than the diameter of the particles, the color of the citrate-coated GNP colloidal solution could change from red to blue; this is due to the resonance band shifting after coupling interactions. Furthermore, the aggregation of nanoparticles is another parameter that contributes to color change. The addition of salt solutions, such as Mg_2_Cl^8^ and NaCl,^9^ can induce a color change from reddish (stable state) to blue (aggregate state) in citrate-coated GNPs. Furthermore, the change in pH can cause GNP aggregation.^10−12^ This color change can be easily observed with the naked eye.^10,13−24^ Unlike quantum dots, GNPs do not require ultraviolet light sources to stimulate various emission colors.

GNPs, with their unique surface plasmon resonance (SPR) and friendly surfaces, have attracted growing interest for use in colorimetric assays for bacterial detection.^25^ The surface of GNPs can be functionalized with several active targeting ligands, such as antibodies, antimicrobial peptides, genetic materials, and thiol-modified ligands.^26−31^ Boronic acid derivatives are recognition molecules that can be used for bacterial detection. They have been widely used to detect bacteria through the binding of boronic acid contained in 4-mercaptophenyl boronic acid (4-MPBA) and *cis*-diol groups in saccharides and glycosylated biomolecules.^32,33^ According to the bacterial structure, peptidoglycan is present in bacterial cell walls. The surface of Gram-negative bacteria is rich in *cis*-diol molecules derived from lipopolysaccharides. Therefore, boronic acids can act as active targeting molecules for the detection of both Gram-negative and Gram-positive bacteria.

**Figure 1:**
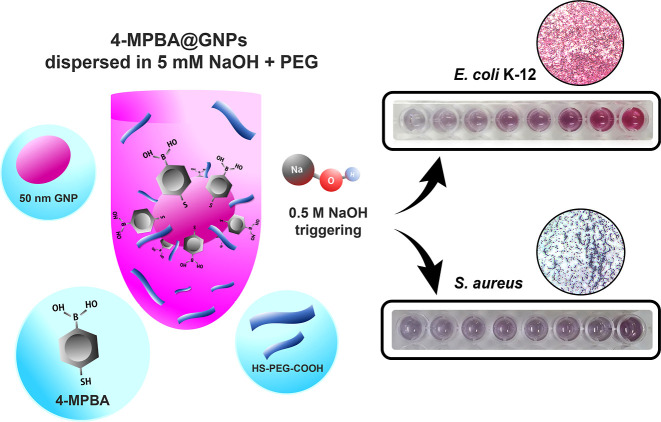
Proposed bacterial detection strategy using 4-MPBA@GNPs in the presence of PEG and 0.5 M NaOH triggering.

In this study, we demonstrate the functionalization of 50 nm GNPs with 4-MPBA, which contains a thiol group (−HS) in its chemical structure. The thiol group of 4-MPBA can functionalize the surface of GNPs via the Au–S reaction.^34^ The 4-MPBA also acts as a bacterial recognition molecule owing to the presence of phenylboronic acid (C_6_H_6_BO_2_) or aromatic boronic acid in its chemical structure. The proposed colorimetric assay for bacterial detection in this study is based on the concentration of bacteria that can inhibit the aggregation of 4-MPBA-functionalized GNPs (4-MPBA@GNPs) in the presence of polyethylene glycol (PEG). PEG was used in this study because of its versatility, ability to increase the stability of nanoparticles, and environmental benignity. The aggregation of 4-MPBA@GNPs in the presence of PEG was mediated by a high concentration of sodium hydroxide (NaOH; 0.5 M). This induction can lead to a color change that can be observed by the naked eye and quantified by using ultraviolet–visible (UV–vis) absorbance spectra. The attachment of 4-MPBA@GNPs to the surface of the bacteria could inhibit aggregation. *Escherichia coli* (*E. coli*) K-12 and *Staphylococcus aureus* (*S. aureus*) were used as Gram-negative and Gram-positive bacterial models in this study, respectively. The color change associated with the NaOH-mediated aggregation of 4-MPBA@GNPs depends on the bacterial concentration and could help distinguish between Gram-negative and Gram-positive bacteria. The complete detection strategy is shown in Figure 1.

## Results and Discussion

### Characterization of Synthesized GNPs and 4-MPBA@GNPs

**Figure 2:**
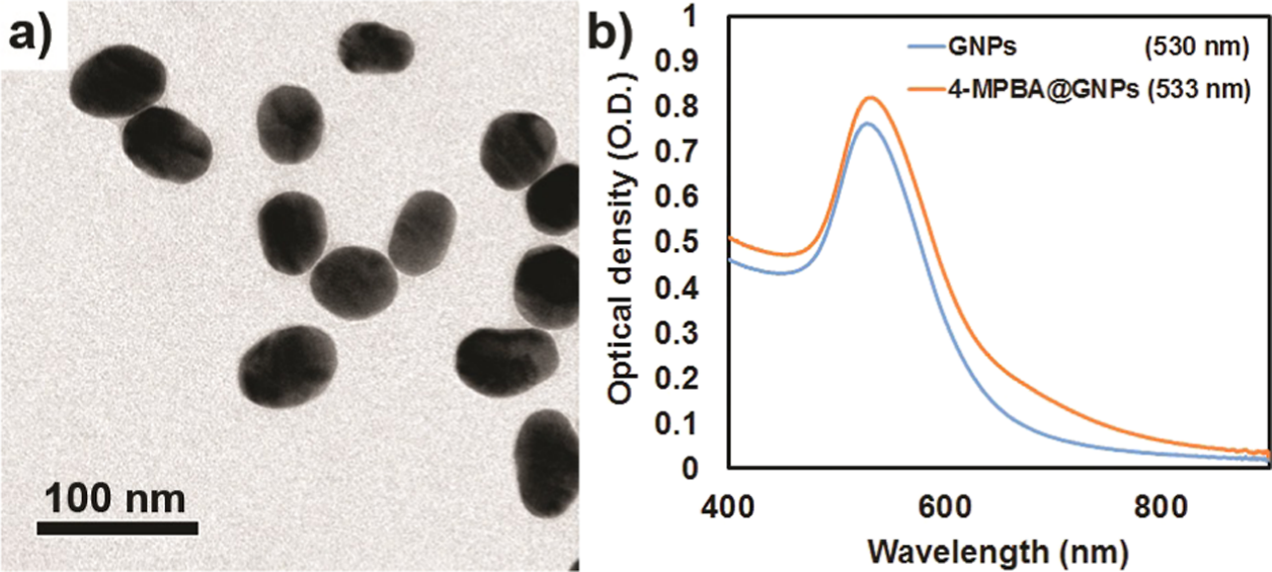
TEM image of (a) GNPs. (b) UV–vis absorption spectra of GNPs and 4-MPBA@GNPs.

The morphology of the GNPs was investigated by using transmission electron microscopy (TEM). The TEM image shows that the GNPs are ellipsoidal in shape (Figure 2a). The GNPs are ∼48.4 ± 0.7 nm in length and 37.9 ± 0.5 nm in width. It is well known that 4-MPBA contains a thiol end group in its structure; therefore, 4-MPBA could be functionalized on the GNP surface through the thiol group.^34^ The functionalization of the surface of GNPs with 4-MPBA was confirmed by measuring the spectra of GNPs and 4-MPBA@GNPs. As shown in Figure 2b, the SPR peak wavelength of the GNPs is 530 nm. However, the SPR peak wavelength of the 4-MPBA@GNPs is ∼533 nm. This red shift at ∼3 nm indicates the functionalization of the surface of the GNPs with 4-MPBA molecules.

The zeta potential or ζ potential and polydispersity index (PdI) values of the GNPs and 4-MPBA@GNPs were also measured. The ζ potential value of 4-MPBA@GNPs (−43.20 ± 0.92 mV) was slightly lower than that of GNPs (−41.83 ± 0.86 mV). When PEG was added to the GNPs and 4-MPBA@GNPs, the ζ potential values increased to −15.43 ± 0.16 and −33.2 ± 0.88 mV, respectively. These results indicate that PEG could interact with the particle surface, resulting in changes in the ζ potential values of the GNPs and 4-MPBA@GNPs. The binding of PEG to the surface of GNPs was due to the reaction between the thiol groups of PEG and GNPs.^35^

**Figure 3:**
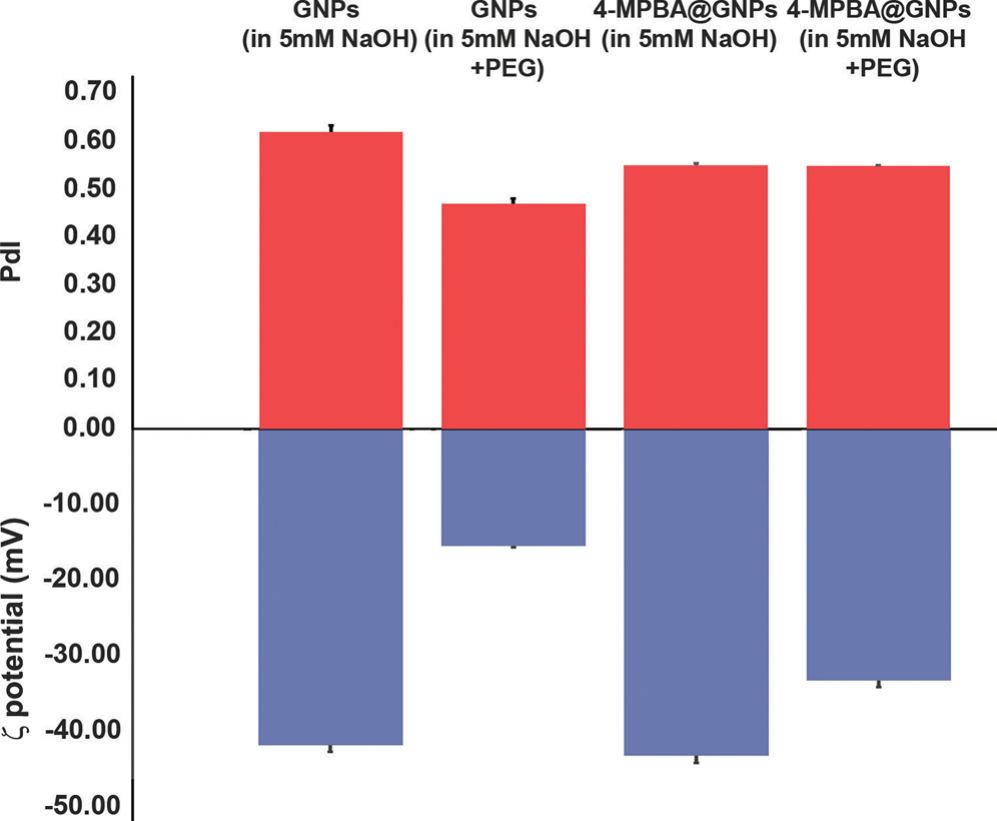
ζ potential and PdI values of GNPs and 4-MPBA@GNPs dispersed in different aqueous media.

PdI values indicate the uniformity and stability of nanoparticles. Values greater than 0.7 imply that very broad particle sizes are distributed in the solution.^36^ Furthermore, these PdI values can indicate the agglomeration or aggregation of nanoparticles in the solution.^37^ As shown in Figure 3, the PdI values of all samples are lower than 0.7. After the modification of the surface of the GNPs with 4-MPBA, the PdI value of 4-MPBA@GNPs decreased from 0.62 ± 0.01 to 0.55 ± 0.002. The change in the PdI values also indirectly reveals the functionalization of the surface of GNPs with 4-MPBA. The addition of PEG molecules to the aqueous media used for the dispersion of 4-MPBA@GNPs resulted in the similar PdI value. However, when GNPs were dispersed in aqueous media containing 0.003 mg mL^–1^ PEG, the PdI value decreased to 0.47 ± 0.01. These data indicate that both 4-MPBA and PEG could help stabilize the surface of GNPs. It is worth noting that all aqueous media containing 5 mM NaOH were used in our experiments. Therefore, we used this condition to measure the PdI and ζ potential values of all GNP forms.

### Impact of 0.5 M NaOH on the ζ Potential Value of 4-MPBA@GNPs

**Figure 4:**
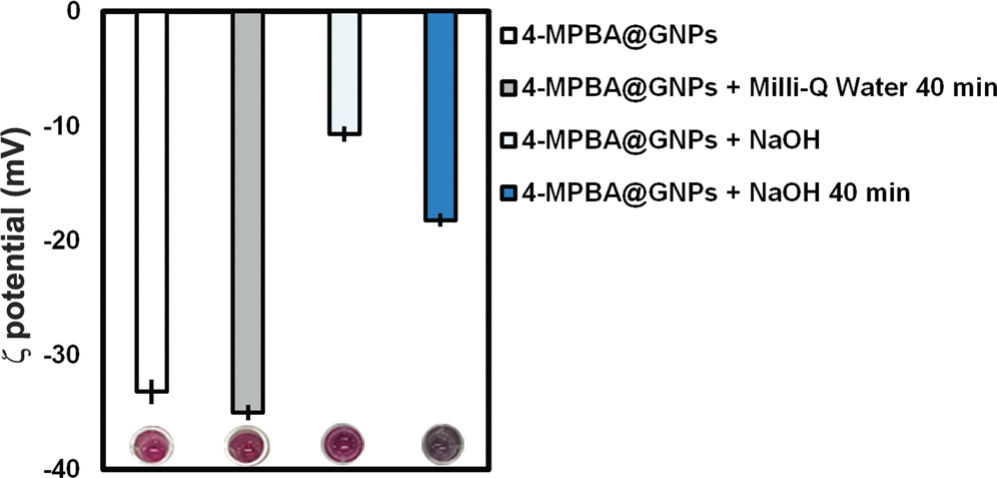
ζ potential values of 4-MPBA@GNPs before and after adding 0.5 M NaOH. The ζ potential values of 4-MPBA@GNPs after adding 0.5 M NaOH and Milli-Q water for 40 min were also measured.

It was previously reported that 4-MPBA molecules could replace the original citrate ions stabilizing on the surface of GNPs functionalized with 4-MPBA molecules.^9,38^ Therefore, the stability of 4-MPBA@GNPs mainly relied on 4-MPBA molecules. However, some triggers can induce the aggregation of 4-MPBA@GNPs. For example, Huang et al.^9^ used a high concentration of sodium chloride (1 M NaCl) to induce the aggregation of 4-MPBA@GNPs. Zheng et al.^39^ demonstrated that an additional amount of 4-MPBA could trigger the aggregation of 4-MPBA@GNPs. In this study, we investigated whether a high concentration of NaOH (0.5 M) affects the ζ potential of 4-MPBA@GNPs dispersed in an aqueous medium containing 5 mM NaOH and PEG. As shown in Figure 4, the ζ potential values of dispersed 4-MPBA@GNPs in the presence of 5 mM NaOH and PEG immediately changed from −33.20 ± 0.88 mV (before adding 0.5 M NaOH) to −10.73 ± 0.40 mV after adding with 0.5 M NaOH. The color of 4-MPBA@GNPs triggered by 0.5 M NaOH also changed to dark purple with the ζ potential value of −18.22 ± 0.39 mV after leaving the suspension of triggered 4-MPBA@GNPs for 40 min. This indicates that 0.5 M NaOH could involve in surface coordination, resulting in the aggregation of 4-MPBA@GNPs. As expected, in the case of 4-MPBA@GNPs added with Milli-Q water and left for 40 min, the ζ potential value (−35.00 ± 0.49 mV) was similar to original 4-MPBA@GNPs (from −33.20 ± 0.88 mV).

### Detection of *E. coli* K-12 and *S. aureus* by Using 4-MPBA@GNPs

**Figure 5:**
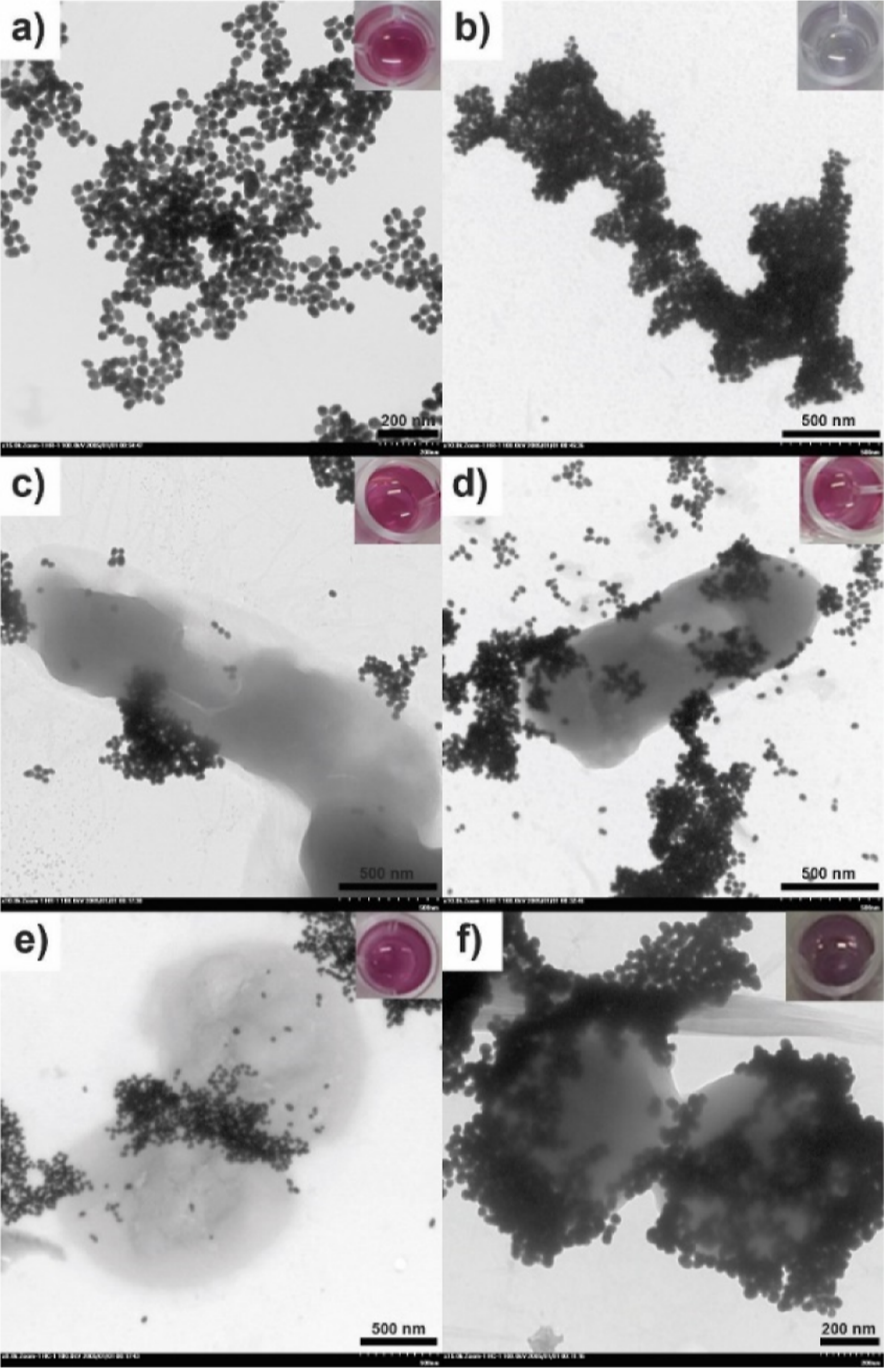
TEM images of 4-MPBA@GNPs under different conditions. (a) 4-MPBA@GNPs dispersed in the solution, (b) 4-MPBA@GNPs dispersed in the solution and 0.5 M NaOH, (c) 4-MPBA@GNPs binding on the surface of *E. coli* K-12, (d) 4-MPBA@GNPs binding on the surface of *E. coli* K-12 and triggered by 0.5 M NaOH, (e) 4-MPBA@GNPs binding on the surface of *S. aureus*, and (f) 4-MPBA@GNPs binding on the surface of *S. aureus* and triggered by 0.5 M NaOH. The concentrations of both bacteria were 2 × 10^7^ CFU mL^–1^.

Two bacterial strains, *E. coli* K-12 and *S. aureus*, were used as model bacteria to investigate the ability of 4-MPBA@GNPs to detect both bacterial strains. As mentioned previously, the 4-MPBA@GNPs used in our system were dispersed in the solution containing 5 mM NaOH and PEG. To the best of our knowledge, this technique has not been reported yet. As seen in Figure 5a, 4-MPBA@GNPs dispersed in the solution containing 5 mM NaOH and PEG appears in red. The TEM image shows a good distribution of spherical 4-MPBA@GNPs. However, when 0.5 M NaOH was added, an aggregation of the 4-MPBA@GNPs occurred. This aggregation was indicated by the color change of the solution from red to blue/pale purple (Figure 5b). In addition, the TEM images show a large cluster arising from the aggregation of 4-MPBA@GNPs after adding 0.5 M NaOH.

When the solution of 4-MPBA@GNPs was added to both bacteria (Figure 5c,e), we found that 4-MPBA@GNPs bound to the surface of *E. coli* K-12 and *S. aureus*. This binding could occur through lipopolysaccharide or peptidoglycan^40^ of *E. coli* K-12 and *S. aureus*, respectively. When 0.5 M NaOH was added to the bacterial samples interacting with 4-MPBA@GNPs and incubated for 40 min, the color of the solution still appeared red for *E. coli* K-12 (Figure 5d) and strong purple for *S. aureus* (Figure 5f). These results strongly confirmed that the binding of 4-MPBA@GNPs on the surfaces of *E. coli* K-12 and *S. aureus* could help resist aggregation that was triggered by 0.5 M NaOH. However, the aggregation prevention/inhibition ability of *S. aureus* was lower than that of *E. coli* K-12.

**Figure 6:**
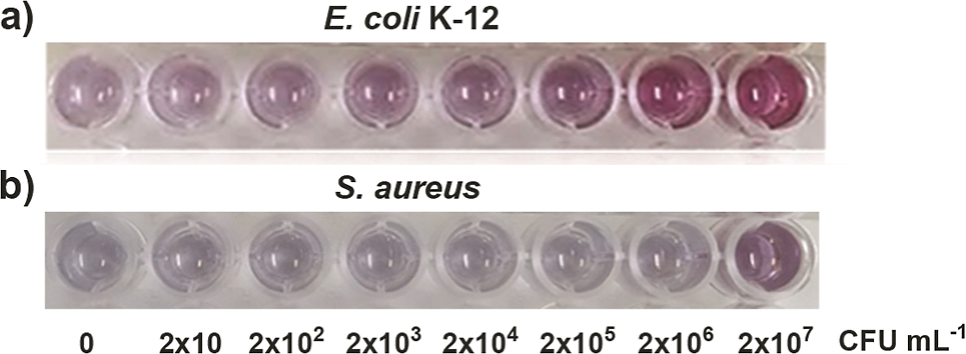
Color appearance of 4-MPBA@GNPs after triggering with 0.5 M NaOH in the samples containing different concentrations of (a) *E. coli* K-12 and of (b) *S. aureus*.

The next step was to investigate the effect of different concentrations of bacteria on the aggregation prevention of 4-MPBA@GNPs. The colorimetric change after adding 4-MPBA@GNPs to *E. coli* K-12 and *S. aureus* at different concentrations, from 0 to 2 × 10^7^ CFU mL^–1^, and then, 0.5 M NaOH was added to trigger the aggregation of 4-MPBA@GNPs, which was observed in the samples. As seen in Figure 6a, the color of the 4-MPBA@GNP solution relied on the concentration of the tested bacteria. The color of the solution without *E. coli* K-12 was pale purple after adding a high concentration of NaOH. The colors of the 4-MPBA@GNP solution were intense when the concentration of *E. coli* K-12 increased. At high concentrations of *E. coli* K-12, the interaction between *E. coli* K-12 and 4-MPBA@GNPs prevented the aggregation of 4-MPBA@GNPs after triggering with 0.5 M NaOH. This resulted in a strong red color in the sample containing *E. coli* K-12 at a concentration of 2 × 10^7^ CFU mL^–1^.

A less intense red color was detected in the sample containing *E. coli* K-12 at a concentration of 2 × 10^6^ CFU mL^–1^. This clearly showed that a good gradient color change was generated in the case of *E. coli* K-12 (Figure 6a). However, in the sample containing *S. aureus*, only the sample containing 2 × 10^7^ CFU mL^–1^*S. aureus* appeared strongly purple. Other samples containing lower concentrations of *S. aureus* showed similar colors (seen as pale purple in Figure 6b) after adding 0.5 M NaOH. This confirms that *E. coli* K-12 could better prevent the aggregation of 4-MPBA@GNPs induced by concentrated NaOH than *S. aureus*.

The mechanism by which a high concentration of NaOH added to the sample containing 4-MPBA@GNPs and bacteria in the presence of PEG could help develop color can be explained. At basic pH, boronic acid can form a reversible complex with *cis*-diol residues, and the environmental pH affects the affinity between boronic acid and *cis*-diol.^32,41^ The color of 4-MPBA@GNPs in the presence of PEG changed from red to pale purple after adding 0.5 M NaOH, indicating the conversion of dispersed 4-MPBA@GNPs and aggregation induction of 4-MPBA@GNPs. The high concentration of NaOH could induce a high ionic strength in the solution of 4-MPBA@GNPs,^42^ which resulted in a reduction in the interparticle distance between 4-MPBA@GNPs^43^ and inducing aggregation of 4-MPBA@GNPs. In the case of the sample containing bacteria at a high concentration of 2 × 10^7^ CFU mL^–1^, the adherence of 4-MPBA@GNPs to bacteria through boronate ester and *cis*-diol on the bacterial surface could help stabilize 4-MPBA@GNPs.^41^ However, the low bacterial concentration was not sufficient to allow adherence between 4-MPBA@GNPs and bacteria. Therefore, some free 4-MPBA@GNPs in the solution might interact with the high ionic strength of 0.5 M NaOH, resulting in aggregated 4-MPBA@GNP formation.^42^ Based on these results, we further investigated the performance of the designed colorimetric probe in the next section.

### Analytical Performance of 4-MPBA@GNPs in the Presence of PEG Interacting with *E. coli* K-12 and *S. aureus* and Then Triggered by 0.5 M NaOH

**Figure 7:**
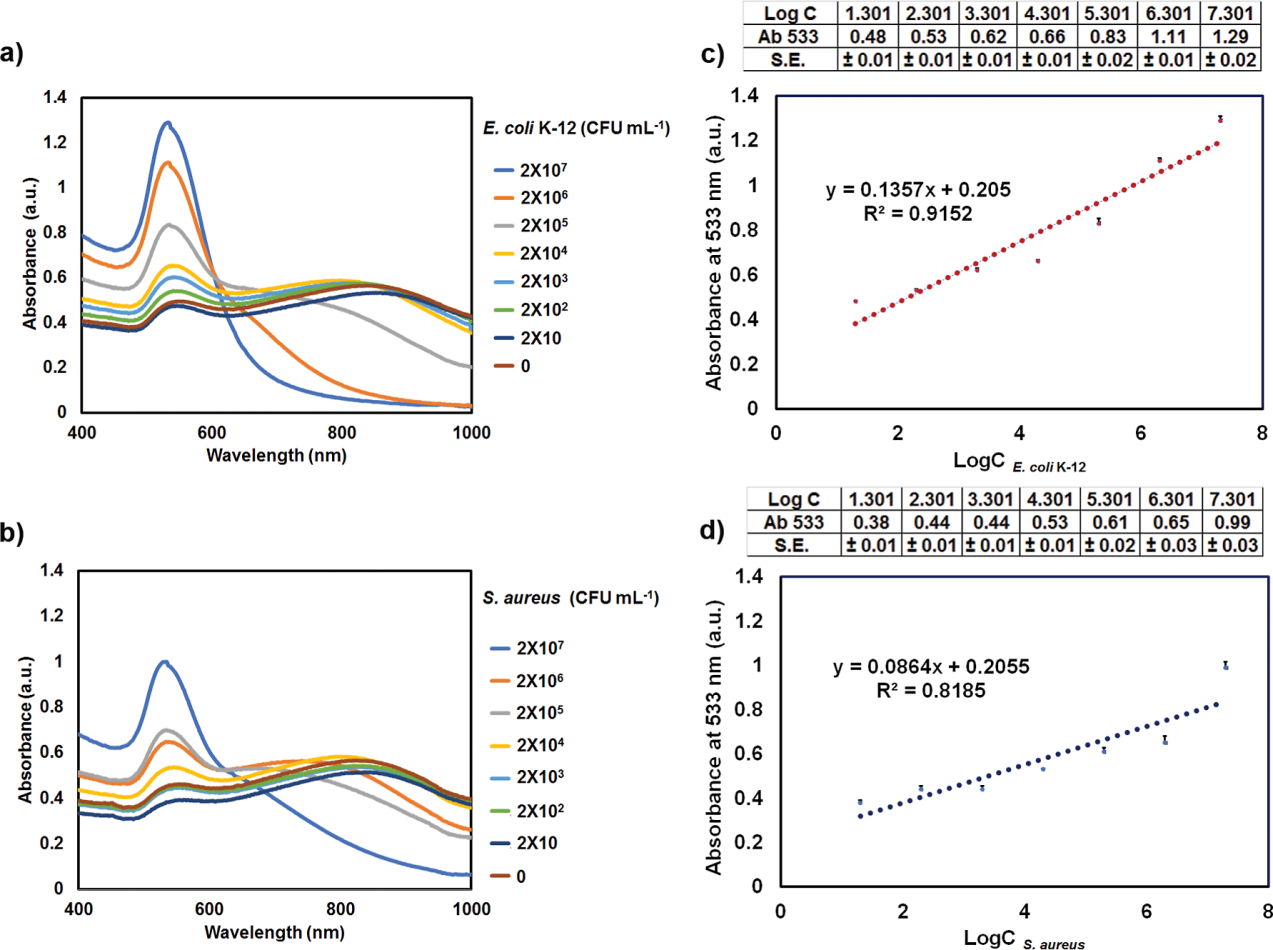
Absorbance spectra of 4-MPBA@GNPs interacting with (a) *E. coli* K-12 and of (b) *S. aureus* with the addition of 0.5 M NaOH and linear response for the detection of (c) *E. coli* K-12 and (d) *S. aureus* with the bacterial concentration ranging from 2 × 10 to 2 × 10^7^ CFU mL^–1^ (*n* ≥ 4).

To evaluate the potential of 4-MPBA@GNPs for the detection of Gram-negative and Gram-positive bacteria using the proposed technique, two bacterial strains at different concentrations were prepared. When 4-MPBA@GNPs interacted with different concentrations of *E. coli* K-12 (0 to 2 × 10^7^ CFU mL^–1^) for 15 min and then 0.5 M NaOH was added to trigger the aggregation of 4-MPBA@GNPs, the absorbance spectra of 4-MPBA@GNPs were investigated. In the samples containing *E. coli* K-12 at concentrations ranging from 0 to 2 × 10^5^ CFU mL^–1^, the second broad absorbance peak appeared at wavelengths of approximately 700–900 nm (Figure 7a). These broad peaks indicate the aggregation of 4-MPBA@GNPs. The broad absorbance peaks of aggregated GNPs were also reported by Tyagi et al.^44^

The sample containing 2 × 10^7^ CFU mL^–1^*E. coli* K-12 had a sharp peak. In the case of the Gram-positive bacteria *S. aureus*, at the same concentration of 2 × 10^7^ CFU mL^–1^, it also shows that only one peak appeared; however, this peak was wider than that of *E. coli* K-12 (Figure 7a,b). The spectra of the mixture of 4-MPBA@GNPs with different concentrations of *E. coli* K-12 or *S. aureus* indicated that the sensitivity of 4-MPBA@GNPs to detect *E. coli* K-12 or *S. aureus* through a 0.5 M NaOH trigger was different.

The UV–vis absorbance of each sample at 533 nm was also measured for quantitatively assessing the detection performance of 4-MPBA@GNPs in the presence of PEG and 0.5 M NaOH. The absorbance of 4-MPBA@GNPs at 533 nm was ∼1.26. After a high concentration of NaOH was used for triggering, the absorbance value of 4-MPBA@GNPs at 533 nm decreased to ∼0.49. In the samples containing *E. coli* K-12 at concentrations of 0 to 2 × 10^7^ CFU mL^–1^, the absorbance intensity at 533 nm gradually increased as the number of *E. coli* K-12 bacteria increased (Figure 7c). A similar trend was also observed in samples containing *S. aureus* (Figure 7d).

The linear relationship between the absorbance intensity and bacterial concentrations of *E. coli* K-12 and *S. aureus* was plotted to evaluate the proposed detection strategy. The limit of detection (LOD) was calculated as LOD = 3.3σ/*S*, where σ is the standard error of the regression slope and *S* is the slope of the calibration curve. We found that the LOD of our proposed approach for *E. coli* K-12 detection was ∼2.38 × 10^2^ CFU mL^–1^ (*R*^2^ = 0.9152, Figure 7c) and ∼4.77 × 10^3^ CFU mL^–1^ for *S. aureus* (*R*^2^ = 0.8185, Figure 7d). *R*^2^ values between 0.7 and 1.0 generally indicate a strong, positive linear relationship.^45^

The color change observed by the naked eye, the absorbance spectra, the *R*^2^ value, and the LOD reveal that our proposed technique has a higher sensitivity for detecting *E. coli* K-12 than *S. aureus*. Although 4-MPBA@GNPs in the presence of PEG could bind to the bacterial cell wall through covalent bonds, as reported by Huang et al.,^9^ the differences in the *cis* diol configuration at the bacterial membrane and ζ potential values of Gram-negative and Gram-positive bacteria can affect the detection performance of our proposed strategy. The change in the environment surrounding nanoparticles, such as surface interruption, aggregation, and the refractive index of the medium, can affect colorimetric changes depending on the aggregation or dispersion of nanoparticles.

Many studies have shown that the binding affinity between boronic acid from 4-MPBA and the *cis* diol configuration of *S. aureus* is higher than that of *E. coli* K-12 owing to the difference in Gram-positive and Gram-negative bacterial surface structures.^46−48^

With our designed detection probe and conditions, the binding of 4-MPBA@GNPs dispersed in PEG to *S. aureus* was higher than that of *E. coli* K-12. This was confirmed by measuring the absorbance of the bacterial pellets after incubation with 4-MPBA@GNPs in the presence of PEG. We found that the absorbance values at a wavelength of 533 nm of the *S. aureus* pellet were ∼2.5 and ∼2.3 for the *E. coli* K-12 pellet. The absorbance values indirectly indicated that 4-MPBA@GNPs were more strongly bound to the surface of *S. aureus* than the surface of *E. coli* K-12. It was reported that the ζ potential values of *E. coli* (∼−44 to −47 mV) were more negative than those of *S. aureus* (∼−35.6 to −38 mV).^49,50^ This is due to the presence of anionic lipopolysaccharide at the outer layer membrane of Gram-negative bacteria.^51^ These different binding sites could affect the color of the reaction solution.

**Figure 8:**
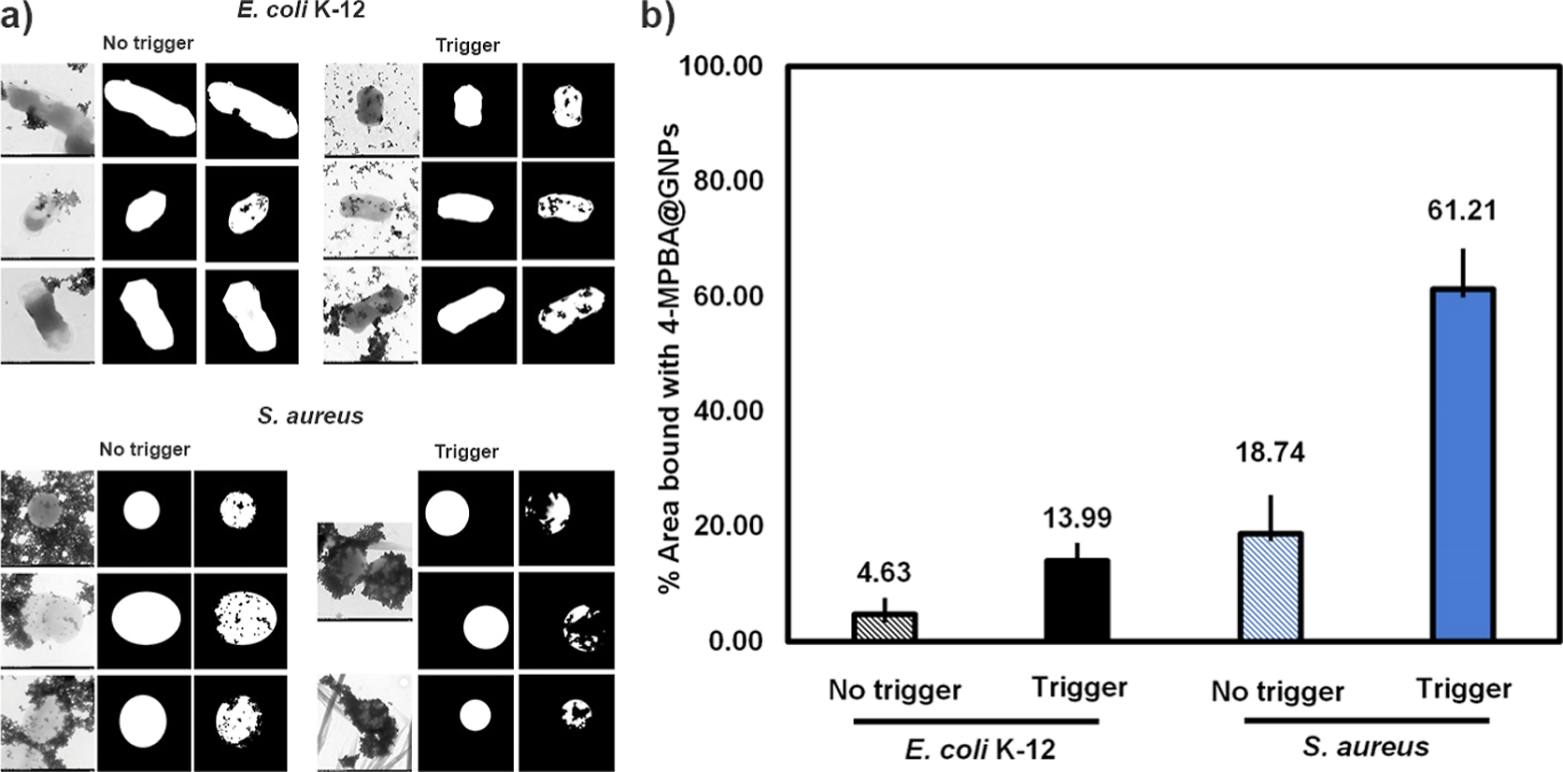
(a) Images and (b) percentages of surface areas of *E. coli* K-12 and *S. aureus* bound with 4-MPBA@GNPs before and after triggering by 0.5 M NaOH.

In addition to the difference in the bacterial membrane, the morphology and size of the bacteria may have an impact on colorimetric appearance. To gain more information, we further investigated the binding of 4-MPBA@GNPs to both bacterial strains. The different amounts of GNPs adhered to the surface of bacteria can depend on the size of the critical volume around the bacterial cells, as reported by Pajerski et al.^52^ As shown in Figure 8, the percentages of bacterial surface areas bound with 4-MPBA@GNPs were different in *E. coli* K-12 and *S. aureus*. All these factors affect the performance of using 4-MPBA@GNPs in the presence of PEG and a high concentration of NaOH.

Our proposed approach for *E. coli* K-12 and *S. aureus* detection could have some benefits when compared to other similar approaches, as shown in Table 1. The proposed 4-MPBA@GNPs and PEG provided a high sensitivity with an LOD of ∼2.38 × 10^2^ CFU mL^–1^ for *E. coli* K-12 when compared to using 4-MPBA@GNPs alone without PEG. Our proposed 4-MPBA@GNPs and PEG also provided different color detections between *E. coli* K-12 and *S. aureus*. However, no significant difference was observed in the color of *E. coli* and other Gram-positive bacteria used in the study by Huang et al.^9^ The other work done by Zheng et al.^53^ provided the LOD of *E. coli* at 1.90 × 10^4^ CFU mL^–1^. Nevertheless, this technique requires the laser irradiation process. It can be found that different techniques have different advantages and disadvantages. In comparison to other similar approaches provided in Table 1, the use of PEG with the 4-MPBA@GNPs could help increase the sensitivity of detection.

**Table 1:** Comparison of Bacterial Detection Using 4-MPBA@GNPs under Different Conditions^a^

	LOD			
probe	*E. coli* (CFU mL^–1^)	*S. aureus* (CFU mL^–1^)	stability	detection time (min)
4-MPBA@GNPs + PEG	∼2.38 × 10^2^	∼4.77 × 10^3^	∼1 week	40
**trigger**: 0.5 M NaOH				
4-MPBA@GNPs^9^	1.02 × 10^3^	NA	NA	20
**trigger**: NaCl				
4-MPBA@GNPs^53^	1.97 × 10^4^	NA	NA	60
**trigger**: 4-MPBA				

a NA = not available.

## Conclusions

This is the first study in which the use of 4-MPBA@GNPs in the presence of PEG and a high concentration of NaOH as an aggregation trigger for detecting Gram-negative (*E. coli* K-12)/Gram-positive (*S. aureus*) bacteria is reported. At the same concentrations of bacteria, the proposed approach can help distinguish between *E. coli* K-12 and *S. aureus* owing to the appearance of different colors. In the case of *E. coli* K-12 detection, a gradient color appears at different concentrations of *E. coli* K-12. This study provides a promising quantitative analytical method for rapid bacterial screening, especially in experimental water that can be contaminated by bacteria. The proposed colorimetric detection method is convenient and suitable for point-of-care testing. Further development of this work can be performed in the future by optimizing the probe condition to overcome the interference from ions or other molecules in tap water or drinking water.

## Experimental Section

### Materials

Hydrogen tetrachloroaurate(III) trihydrate (HAuCl_4_·3H_2_O, 99.9%, 2 g L^–1^), trisodium citrate (Na_3_C_6_H_5_O_7_·2H_2_O), 4-MPBA [HSC_6_H_4_B(OH)_2_], thiol–PEG–carboxyl (HS–PEG7500–COOH), and NaOH were purchased from Sigma-Aldrich (St. Louis, MO, USA). Nutrient broth (NB) and agar powder were purchased from HIMEDIA (Mumbai, India).

### Synthesis of GNPs

GNPs having a size of ∼50 nm were synthesized by the citrate reduction of HAuCl_4_, known as Turkevich’s method. First, 5 mL of HAuCl_4_·3H_2_O (99.9%, 2 g L^–1^) was added to 95 mL of Milli-Q water. The solution was heated and stirred until the temperature reached 95 °C. At this temperature, 1.8 mL of 19.4 mM tri-sodium citrate was immediately added to the HAuCl_4_ solution and vigorously stirred. The solution was continuously heated and stirred until a dark pink color was observed. Subsequently, the solution was heated and stirred for another 30 min. Then, the heat was removed, and the mixture was continuously stirred for 30 min. The synthesized 50 nm GNPs were stored at 4 °C until use.

### Functionalization of GNPs with 4-MPBA

The surface of 50 nm GNPs was functionalized with 4-MPBA by centrifuging the nanoparticles at 1100*g* for 30 min. After centrifugation, the supernatant was removed, and the pellet was dispersed in Milli-Q water. The optical density (OD) of the GNP solution at 530 nm (SPR wavelength of the prepared 50 GNPs) was adjusted to 1.0 before functionalization was performed. Following this, 13.5 mL of GNPs was mixed with 0.3 mL of 4-MPBA (2 mg mL^–1^; dissolved in 0.2 M NaOH), and the mixture was shaken for 12 h. Then, the mixed solution was centrifuged twice at 1100*g* for 40 and 30 min. The pellet from the first round of centrifugation was dispersed in 5 mM NaOH, and the pellet from the final round of centrifugation was dispersed in 5 mM NaOH containing 0.003 mg mL^–1^ of HS–PEG–COOH (*M*_n_ ∼ 7.5 kDa). The reason why 5 mM NaOH was chosen is that it can help maintain the stability of 4-MPBA@GNPs. The final particles from the functionalization between the GNPs and 4-MPBA were named 4-MPBA@GNPs. The suspension of 4-MPBA@GNPs was stored at 4 °C until further use. The absorption spectrum of the 4-MPBA@GNPs was measured by using UV–vis spectroscopy to detect the SPR peak wavelength of the 4-MPBA@GNPs.

### Characterization of GNPs and 4-MPBA@GNPs

The synthesized GNPs were characterized by using UV–vis spectroscopy, and their morphologies were investigated by using TEM. The ζ potentials of the synthesized GNPs and 4-MPBA@GNPs were measured using a Zetasizer (Malvern, UK). GNPs and 4-MPBA@GNPs were dispersed in Milli-Q water, 5 mM NaOH, and 5 mM NaOH containing 0.003 mg mL^–1^ HS–PEG–COOH. All dispersed GNPs and 4-MPBA@GNPs were adjusted to obtain an OD of 1.0 at their SPR wavelength before measuring the ζ potential values. The PdI of all samples was also measured.

To investigate the morphologies of GNPs and 4-MPBA@GNPs, a droplet of the solution of GNPs or 4-MBPA@GNPs was placed on a copper grid coated with formvar, and the samples were dried. TEM was used to observe the morphologies of GNPs and 4-MPBA@GNPs. TEM images were also used to measure the percentages of bacterial surface areas bound with 4-MPBA@GNPs by ImageJ software.

### Preparation of Bacteria

*E. coli* K-12 (ATCC 10798) and *S. aureus* (ATCC 25923) were grown in NB and stored in an incubator at 37 °C overnight. The bacterial suspension was centrifuged twice at ∼1900*g* for 10 min to remove the NB. The obtained bacterial pellet was then resuspended in Milli-Q water and adjusted to an OD of 0.5 at 600 nm. Serial dilutions of 10^–1^, 10^–2^, 10^–3^, 10^–4^, 10^–5^, 10^–6^, and 10^–7^ were prepared for *E. coli* K-12 or *S. aureus*.

To identify the number of bacteria (colony-forming units per mL; CFU/mL), 100 μL of each diluent was dropped on the nutrient agar, and the spread-plating technique was applied. The plates were then incubated overnight at 37 °C. After incubation, the number of bacteria was counted, and CFU mL^–1^ was calculated using the following equation.
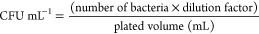


### Colorimetric Assay

The solution of 4-MPBA@GNPs dispersed in 5 mM NaOH containing 0.003 mg mL^–1^ HS–PEG–COOH (PEG) was adjusted to have an OD of 3.0 at 533 nm (SPR peak wavelength of prepared 4-MPBA@GNPs). As previously mentioned, the bacteria used as target models in this study were *E. coli* K-12 and *S. aureus*. Bacteria were detected by adding 100 μL of 4-MPBA@GNP solution to wells containing bacteria (*E. coli* K-12 or *S. aureus*) at different concentrations from 0 to 2 × 10^7^ CFU mL^–1^. The final volume of the solution in the well was 200 μL, and the final concentration of bacteria in the final mixture ranged from 0 to 1 × 10^7^ CFU mL^–1^. The mixture was then mixed and incubated for 15 min. Next, 25 μL of 0.5 M NaOH was immediately added to the mixture to induce the aggregation of 4-MPBA@GNPs. Thereafter, the sample was mixed thoroughly and incubated for 30–45 min at room temperature. After incubation, a reaction based on a change in color was observed. The UV–vis absorbance of the reaction mixture was monitored by measuring the OD at a wavelength of 533 nm. The mean OD value was calculated from the different sets of experiments. The linear relationship between the absorbance intensity at 533 nm and bacterial concentration was plotted. The LOD was calculated as LOD = 3.3σ/*S*, where σ is the standard error of the regression slope and *S* is the slope of the calibration curve.^36^
